# Changes in severity, mortality, and virus genome among a Spanish cohort of patients hospitalized with SARS-CoV-2

**DOI:** 10.1038/s41598-021-98308-x

**Published:** 2021-09-22

**Authors:** Rocío Aznar-Gimeno, J. Ramón Paño-Pardo, Luis M. Esteban, Gorka Labata-Lezaun, M. José Esquillor-Rodrigo, Angel Lanas, David Abadía-Gallego, Francisco Diez-Fuertes, Carlos Tellería-Orriols, Rafael del-Hoyo-Alonso, M. Trinidad Serrano

**Affiliations:** 1 Department of Big Data and Cognitive Systems, Instituto Tecnológico de Aragón, ITAINNOVA, María de Luna 7-8, 50018 Zaragoza, Spain; 2 Infectious Disease Department, University Clinic Hospital Lozano Blesa, San Juan Bosco 15, 50009 Zaragoza, Spain; 3 University of Zaragoza, Zaragoza, Spain; 4 Aragon Health Research Institute (IIS Aragon), Zaragoza, Spain; 5 Escuela Universitaria Politécnica de La Almunia, Universidad de Zaragoza, Calle Mayor, 5, 50100 La Almunia de Doña Godina, Zaragoza, Spain; 6 Internal Medicine Department, University Clinic Hospital Lozano Blesa, San Juan Bosco 15, 50009 Zaragoza, Spain; 7 Service of Digestive Diseases, University Clinic Hospital Lozano Blesa, San Juan Bosco 15, 50009 Zaragoza, Spain; 8 CIBEREHD, Zaragoza, Spain; 9 AIDS Immunopathology Unit, Centro Nacional de Microbiología, Instituto de Salud Carlos III, Ctra. Majadahonda-Pozuelo, Km. 2, 28220 Majadahonda, Madrid, Spain; 10 Hospital Clínic-Institut d’Investigacions Biomèdiques August Pi I Sunyer (IDIBAPS), 08036 Barcelona, Spain; 11 Biocomputing and Health Data Science, Instituto Aragonés de Ciencias de La Salud (IACS), San Juan Bosco 13, 50009 Zaragoza, Spain

**Keywords:** Risk factors, Viral infection

## Abstract

Comparing pandemic waves could aid in understanding the evolution of COVID-19. The objective of the present study was to compare the characteristics and outcomes of patients hospitalized for COVID-19 in different pandemic waves in terms of severity and mortality. We performed an observational retrospective cohort study of 5,220 patients hospitalized with SARS-CoV-2 infection from February to September 2020 in Aragon, Spain. We compared ICU admissions and 30-day mortality, clinical characteristics, and risk factors of the first and second waves of COVID-19. The SARS-CoV-2 genome was also analyzed in 236 samples. Patients in the first wave (n = 2,547) were older (median age 74 years [IQR 60–86] vs. 70 years [53–85]; p < 0.001) and had worse clinical and analytical parameters related to severe COVID-19 than patients in the second wave (n = 2,673). The probability of ICU admission at 30 days was 16% and 10% (p < 0.001) and the cumulative 30-day mortality rates 38% and 32% in the first and second wave, respectively (p = 0.007). Survival differences were observed among patients aged 60 to 80 years. We also found some variability among death risk factors and the viral genome between waves. Therefore, the two analyzed COVID-19 pandemic waves were different in terms of disease severity and mortality.

## Introduction

COVID-19, caused by SARS-CoV-2, is the first major pandemic humankind has faced in over 100 years. Pursuing naturally acquired herd immunity is not a feasible strategy^1^. In contrast to SARS-CoV-1, the transmission of SARS-CoV-2 is expected to resemble that of pandemic influenza, with several pandemic waves, followed by seasonal circulation as may have previously happened with other known coronaviruses^2,3^. We do not yet know how far this virus will continue to be transmitted. The emergence of specific, highly effective vaccines offers hope for the future^4–7^, but only universal vaccination will prevent further outbreaks.

Although there have been different pandemic waves, no extensive studies have analyzed whether the severity and mortality are similar, or whether there are variations depending on the different situations in which they arise.

Aragón is an autonomous Spanish community that experienced the first pandemic wave between February and May 2020. After a period of full lockdown, community transmission of SARS-CoV-2 decreased markedly^8^. After several local outbreaks in June and July, SARS-CoV-2 transmission was widespread, and Aragón had the highest midsummer incidence in the European region^9,10^. Although there are indications that the second wave was less severe than the first^11^, data supporting this hypothesis are scarce. Similarly, although studies have identified risk factors for disease severity^12^, studies comparing the evolution of hospitalized patients in terms of severity and mortality are lacking.

The present study compared the two first different pandemic waves in terms of severity and mortality (ICU admission and death) among hospitalized patients with COVID-19 and analyzed the characteristics of the affected population and risk factors for severity. This knowledge can help us understand the behavior of this disease over time.

## Methods

### Design and setting

This is an observational retrospective cohort study that includes all patients hospitalized with COVID-19 in Aragon, a region in Northeastern Spain that comprises 1,328,753 inhabitants (January 1, 2020) with a highly centralized distribution; 52.7% of the population lives in one city. The publicly funded healthcare system (SALUD) covers the entire population of the region via seven hospitals. Two of these hospitals are university hospitals with more than 700 beds, and the rest are regional hospitals.

The research protocol was approved by the Clinical Research Ethics Committee of Aragon (PI 20/183) according to good clinical practices and applicable laws and regulations. Due to the retrospective, observational nature of this study, the data could be fully anonymized and informed consent was waived. All methods were performed in accordance with the relevant guidelines and regulations.

### Patients and data acquisition

Our primary data source was the Aragón Healthcare Records Database, which we accessed through the *BIGAN Gestion Clinica* platform of the Aragón Department of Health. This database contains demographic and clinical information on all individuals covered by SALUD.

COVID-19 diagnosis was confirmed by positive SARS-CoV-2 RT-PCR. Laboratory-confirmed COVID-19-associated hospitalizations were identified using laboratory and electronic medical records databases. Hospitalization was considered chronologically related to COVID-19 when it occurred within the first 20 days after or no more than 10 days before the first positive SARS-CoV-2 PCR test.

Data were extracted for a total of 5,220 patients with SARS-CoV-2 infection who were hospitalized in the SALUD hospital network between February 27, 2020, and September 23, 2020.

The criteria for admission and hospital management of patients with SARS-Cov-2 infection are based on the recommendations contained in the technical document published by Spanish Ministry of Health. The document includes *Covid-19 emergency management* and *Covid-19 clinical management: hospital care*^13^. Each hospital has its own protocol adapting these recommendations to its characteristics. The information is available at the websites for each hospital. The hospitalization and ICU admission criteria, according to these guidelines, have remained stable throughout the pandemic. Ultimately, however, clinical decisions may vary at the discretion of practitioner.

**Figure 1:**
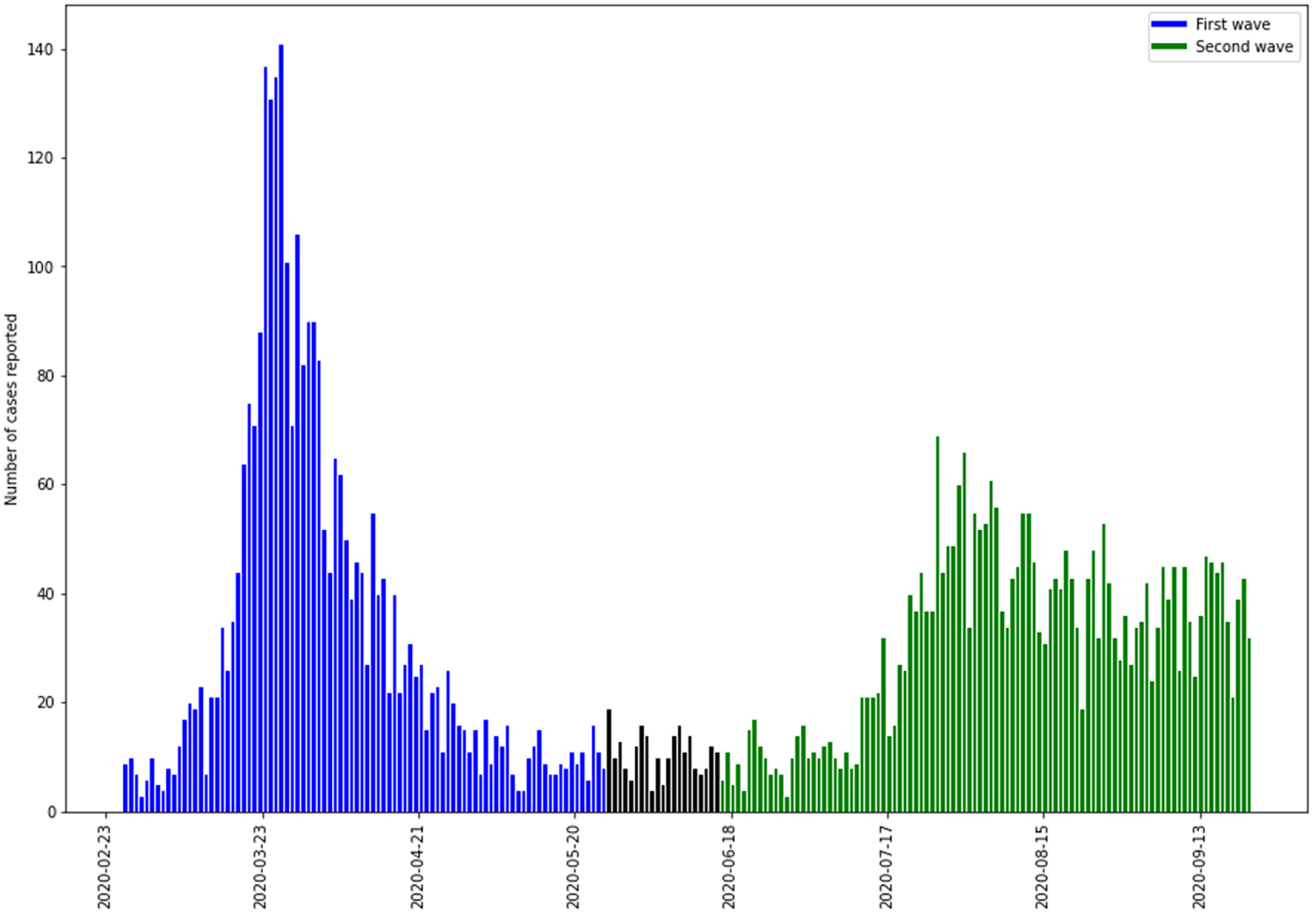
Hospitalized patients with positive SARS-Co V-2 RT-PCR test results in both pandemic waves in Aragon, Spain, February-September 2020. *Blue*: first wave; *green*: second wave.

The beginning and end of each wave was defined based on the variation in SARS-CoV-2 infection incidence observed from February 27 to September 23. We observed two well-defined waves: the first from February 27 to May 27, 2020, and the second from June 17 to September 23, 2020 (Fig. 1). However, by September 23, the second wave has not fully declined.

We collected data on patient demographics, comorbidities, and drugs prescribed in the 6 months prior to hospitalization, Vital signs were recorded upon arrival at the emergency room for all patients, Laboratory variables measured in the first 24 h were available only for the largest two hospitals in the SALUD network, which represented 60.6% of all patients admitted with COVID-19 in the region. The observation period lasted up to 30 days after the last patient was included in the analysis. The main outcomes were admission into the intensive care unit (ICU) and 30-day all-cause mortality after hospital admission.

The study followed the Strengthening the Reporting of Observational Studies in Epidemiology (STROBE) guidelines for cohort studies^14^.

### Statistical analysis

Our study compared four main topics between the two waves: descriptive variables, mortality risk factors, severity and mortality (longitudinal analysis), and the viral genome.

#### Descriptive analysis

We performed a comparative descriptive analysis of the first- and second-wave cohorts stratified by hospitalized, ICU admission, and death. Previously, normality was tested using the Shapiro–Wilk test. As continuous variables failed the normal distribution, they are presented as the median and interquartile range (IQR), and categorical variables are presented as absolute and relative frequencies. Comparisons were performed using the Mann–Whitney test for continuous variables and chi-squared test for categorical variables or proportions.

#### Identification of mortality risk factors

We analyzed univariate logistic regression models to identify predictors of death in each of the subcohorts. To better identify the truly significant predictors in the univariate analysis, not based only on an extensive search, we estimated the adjusted p-values for multiple comparisons using the Holm method^15^.

Multivariate analysis provides additional information about which of these predictors are independent risk factors. To analyze the difference between waves, we have defined a new dichotomical categorical variable that distinguish between waves. Logistic regression model has been constructed taking as candidates not only the predictor variables, but also their interaction with the variable defining the wave Thus, if the interaction term is statistically significant we can affirm that we found differences for this predictor variable between waves.

The discriminatory capacities of univariate significant risk factors were evaluated by measuring the area under the receiver operating characteristic (ROC) curve (AUC). Results are expressed as odds ratios (ORs) and p-values.

#### Longitudinal analysis of severity and mortality

Two longitudinal analyses were performed in the cohort of hospitalized patients to evaluate the management of ICU admission and occurrence of death in the two waves. The follow-up time started from the hospitalization date and ended at either the ICU admission date or the discharge/death date. We used cumulative incidence curves to analyze the longitudinal data, and the Gray test to compare variables between waves for groups stratified by sex or age.

The threshold p-value was set at 0.05. Analyses were performed using R version 3.6.2 language programming (R Foundation for Statistical Computing, Vienna, Austria) and Python version 3.7 provided by Jupyter (jupyter.org). R statistical software was used mainly for statistical analysis and Python for data retrieval and preparation.

#### Viral genome analysis

Whole genome sequences (> 29,000 bp) of SARS-CoV-2 from Aragon (n = 295) were retrieved from the database established by the global initiative on sharing all influenza data (GISAID). We evaluated how these sequences were distributed among different phylogenetic clades. Sequences were aligned by an iterative refinement method implemented in MAFFT version 7 software^16^ and manually edited in Bioedit v7.2.5^17^. The phylogeny of the alignment was inferred with IQ-Tree software v2.1.1^18^, and node support was assessed by an ultrafast bootstrap approximation. The TIM2 substitution model, with unequal base frequencies and a proportion of invariant sites (TIM2 + F + I), was selected as the best-fit model according to the Bayesian information criterion. In Aragón, the prevalence of the D614G mutation was tracked throughout the pandemic by aligning this region in all 295 genomes.

## Results

### Descriptive analysis

**Figure 2:**
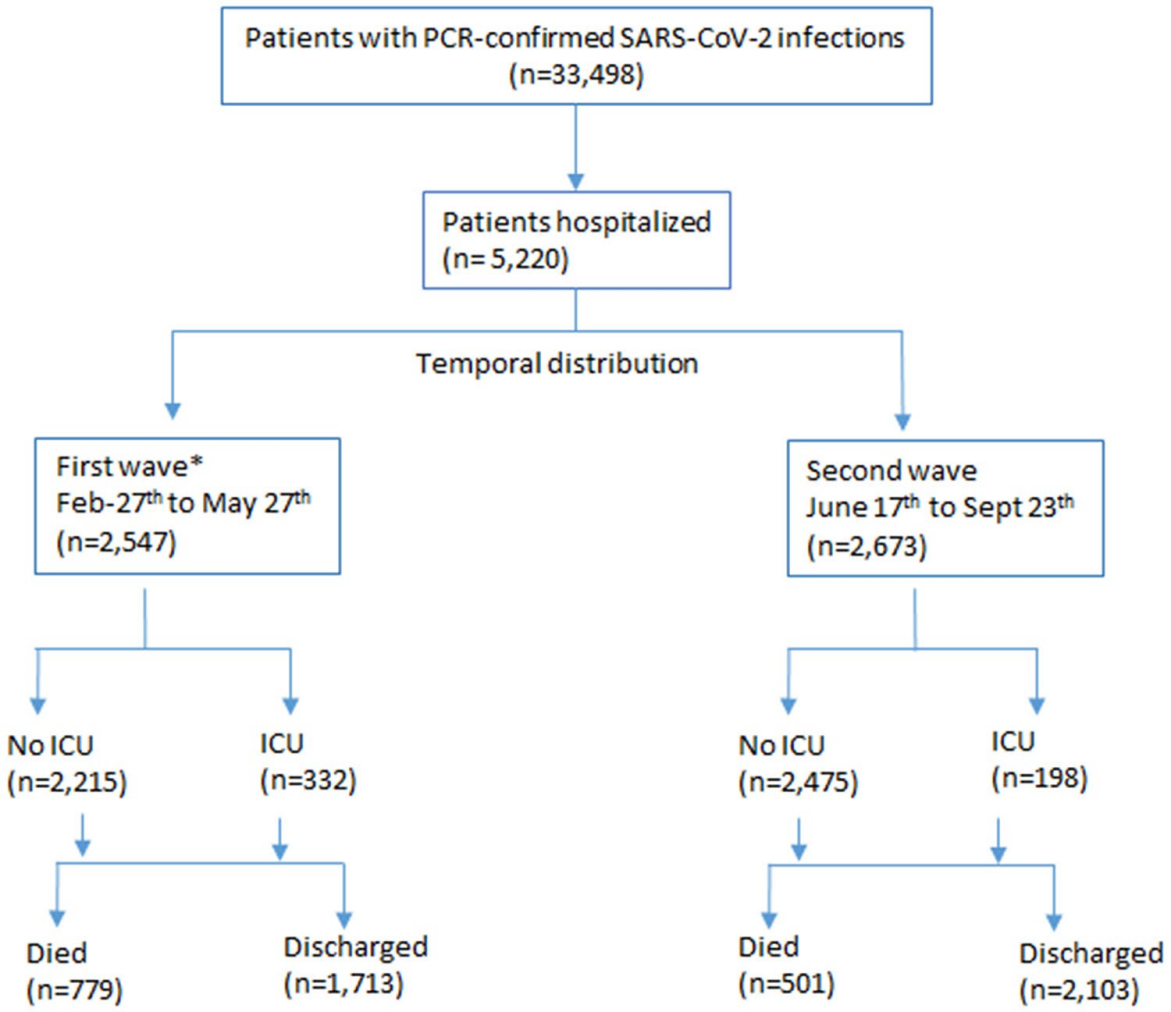
Selection and analysis of study participants in Aragon, Spain, February-September 2020.

**Figure 3:**
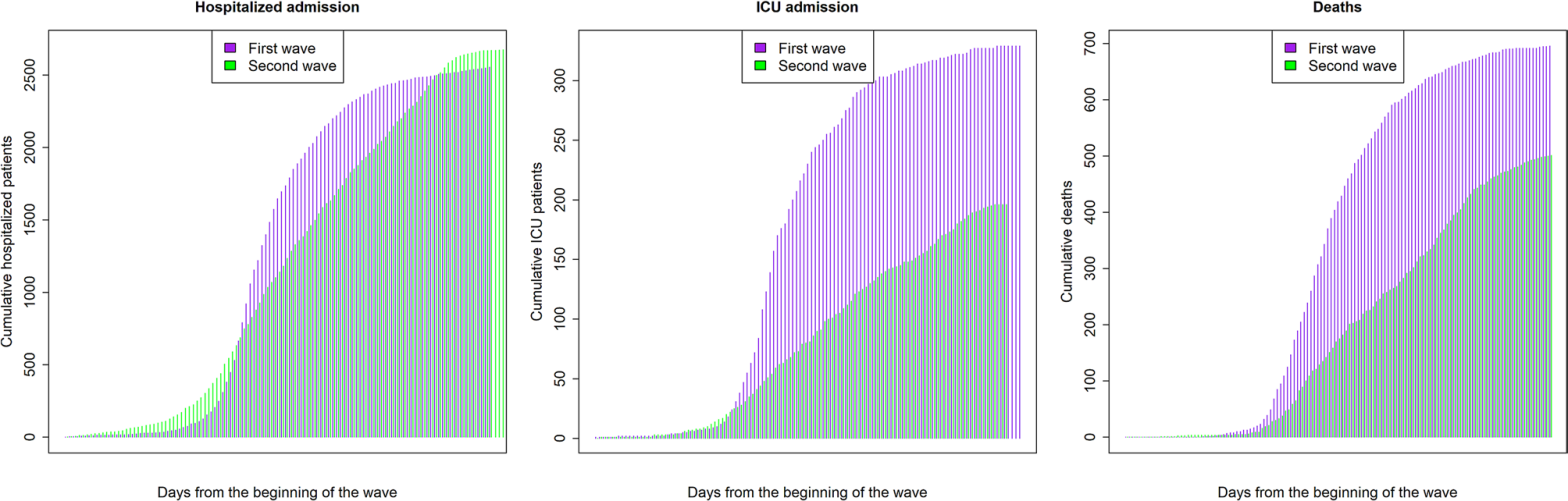
Comparison of cumulative cases between pandemic waves in Aragon, Spain, February-September 2020. Prevalence of SARS-CoV-2 cases confirmed by RT-PCR during the first (*purple*) and second waves (*green*) are shown for hospitalized patients (*left panel*), ICU admissions (*middle panel*), and deaths (*right panel*). ICU: intensive care unit.

The patient flowchart is given in Fig. 2. A total of 2,547 patients were hospitalized with SARS-CoV-2 infection in the first wave and 2,673 in the second wave. Of these patients, 332 (13%) and 198 (7.4%) were admitted to the ICU (p < 0.001), and 779 (30.6%) and 501 (18.7%) died (p < 0.001) during the first and second waves, respectively. The cumulative cases between waves are compared in Fig. 3.

**Figure 4:**
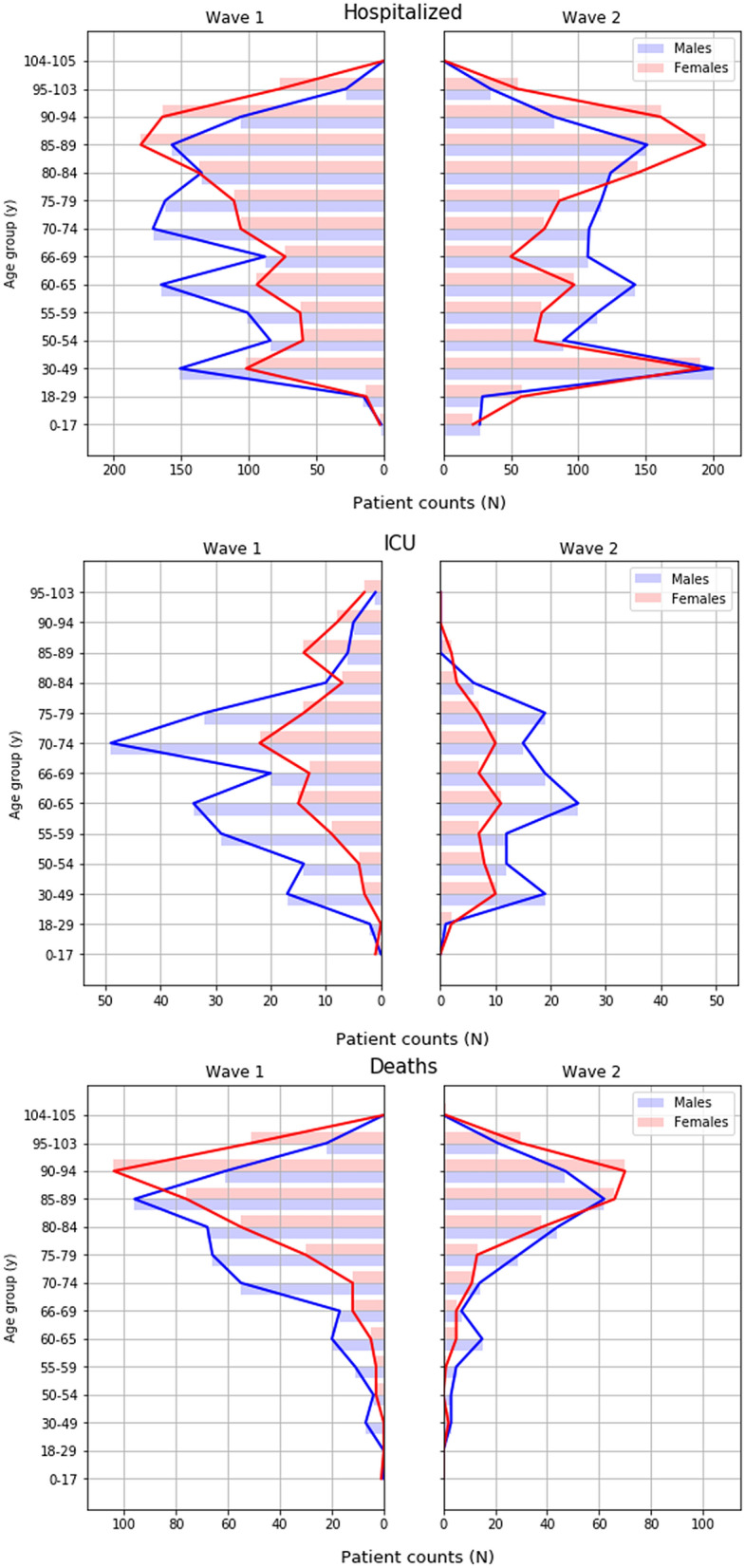
Sex and age distributions of patients who tested positive for COVID-19 during the two pandemic waves in Aragon, Spain, February-September 2020. ICU: intensive care unit. *Top*: Hospitalized patients; *Middle*: patients admitted to ICU; *Bottom*: patients who died. *Blue*: males; *red*: females.

The age and sex distributions of patients who were hospitalized, admitted to the ICU, or deceased are shown for both waves in Fig. 4. In both waves, a higher proportion of men were hospitalized with COVID-19 compared to women. This difference increased in the population admitted to the ICU, but not in regards to mortality. However, for each of these subpopulations (hospitalized, ICU admission, death), the sex distribution was not significantly different between waves (p > 0.927).

Among all patients hospitalized with COVID-19, the mean age was significantly different between the two waves (Table 1). Patients in the first wave were older (median 74 years, [IQR 60–86] vs. 70 years [53–85]; p < 0.001) and had more comorbidities, including cerebrovascular disease and dementia, as well as previous pneumonia than patients in the second wave. However, the second wave had a higher frequency of diabetes. In addition, previous drug treatments in hospitalized patients differed between the two pandemic waves.

**Table 1:** Comparisons of clinical and laboratory variables between the two COVID-19 pandemic waves, Aragon, Spain, February–September 2020.

Variable	Hospitalized patients			ICU patients			Deaths		
	First wave	Second wave	p-value	First wave	Second wave	p-value	First wave	Second wave	p-value
Sex Male/Female n (%)	1365 (53.6)/1182 (46.4)	1372 (51.3)/1301 (48.7)	0.097	219 (66.0)/113 (34.0)	131 (66.2)/67 (33.8)	1.000	427 (54.8)/352 (45.2)	256 (51.1) 245 (48.9)	0.207
Age (y)	74 (60–86)	70 (53–85)	< 0.001	70 (60–76)	62 (54–72)	< 0.001	85 (77–90)	87 (81–91)	0.011
**Emergency room**									
Systolic pressure (mm Hg)	126 (112–141)	128 (114–142)	0.126	127 (114–143)	130 (115–144)	0.319	122 (108–141)	129 (113–145)	0.008
Diastolic pressure (mm Hg)	71 (62–80)	72 (64–80)	0.111	73 (63–81)	73.5 (66–81)	0.210	68 (59–78)	69 (61–78)	0.298
Heart rate (bpm)	86 (75–98)	83 (73–97)	0.001	90 (78–101)	88 (77–100)	0.265	87 (74–100)	85 (73–98)	0.183
Respiratory rate (bpm)	25 (20–32)	26 (22–32)	0.487	26 (20.5–32 )	27.5 (23.7–32)	0.329	30 (24–34)	31 (24–36)	0.194
Temperature (ªC)	36.8 (36.3–37.5)	36.6 (36.2–37)	< 0.001	37 (3.5–37.8 )	36.7 (36.3–37.3)	0.001	36.9 (36.3–37.5)	36.6 (36.3–37.2)	0.001
Oxygen saturation (%)	95 (92–97)	95 (93–97)	< 0.001	94 (90–97)	94 (92–96)	0.374	94 (90–96)	94 (92–96)	0.004
Oxygen treatment (%)	278 (12.7)	239 (9.6)	0.001	41 (13.7)	18 (10)	0.262	75 (11.9)	50 (10.5)	0.499
Capillary blood glucose (mg/dl)	147 (118–194)	150 (121–215)	0.329	147 (117.7–195.5)	151 (126–210)	0.590	162 (124.5–218)	180 (140–255)	0.056
**Laboratory**									
Glucose (mg/dl)	113 (97–139)	116 (97–144)	0.434	156.5 (117–211)	164 (123–203)	0.481	123 (101–164)	132 (104–172)	0.105
Creatinine (mg/dl)	0.94 (0.74–1.29)	0.89 (0.69–1.19)	< 0.001	0.93 (0.71–1.31)	0.73 (0.58–0.99)	< 0.001	1.22 (0.91–1.86)	1.18 (0.84–1.64)	0.091
Urea (g/l)	0.421 (0.3–0.69)	0.4 (0.29–0.62)	0.003	0.51 (0.36–0.74)	0.47 (0.34–0.62)	0.123	0.71 (0.5–1.06)	0.63 (0.43–0.94)	0.014
Chloride (mmol/l)	101 (98–105)	102 (99–105)	0.032	103 (99–106)	104 (102–107)	< 0.001	103 (99–107)	102 (99–106)	0.412
Potassium (mmol/l)	4.17 (3.84–4.53)	4.19 (3.86–4.52)	0.904	4.14 (3.77–4.59)	4.11 (3.85–4.41)	0.561	4.31 (3.84–4.68)	4.25 (3.83–4.59)	0.241
Ionic Calcium (mmol/l)	1.17 (1.13–1.22)	1.15 (1.11–1.19)	< 0.001	1.13 (1.08–1.17)	1.13 (1.09–1.17)	0.812	1.18 (1.13–1.22)	1.16 (1.11–1.2 )	0.001
Alanine aminotransferase (ALT) (U/l)	23 (15–40)	24 (15–40)	0.953	36 (23–58)	35 (21–49)	0.227	20 (13–32)	19 (13–29.5)	0.289
Aspartate aminotransferase (AST) (U/l)	33 (24–49.75)	32 (23–47)	0.235	47 (31–74.5)	38 (27–54)	0.004	35 (23–53)	34 (24–49)	0.610
Lactate dehydrogenase (LDH) (U/l)	292 (230–398 )	286 (224–372 )	0.018	465 (345–600 )	438 (332–546)	0.247	319 (244–439)	310 (238–458)	0.609
Prothrombin activity (APT) (%)	85 (74–96)	90 (78–102)	< 0.001	81 (68.5–94)	83 (73–97.75)	0.077	81 (66–91)	84 (71–99.5)	0.004
International normalized ratio-prothrombin time (INR-PT)	1.12 (1.06–1.23)	1.08 (1.02–1.18)	< 0.001	1.16 (1.07–1.29)	1.13 (1.05–1.24)	0.027	1.17 (1.08–1.36)	1.13 (1.03–1.26)	0.001
Active partial thromboplastin time (RATIO-APTT) (seconds)	1 (0.91–1.1)	0.97 (0.89–1.06)	< 0.001	0.97 (0.9–1.08)	0.91 (0.83–0.99)	< 0.001	1.01 (0.92–1.11)	0.97 (0.89–1.08)	0.003
D-Dimer (microgr/l)	958 (519–1770)	758 (428–1431)	< 0.001	1334 (808–2418)	1129 (612–2567)	0.310	1495 (972–3603)	1327 (772–3893)	0.139
Fibrinogen (mg/dl)	700 (599–709)	657 (550–700)	< 0.000	700 (657–847)	700 (577–764)	0.004	680 (571–715)	650 (531–700)	0.008
Leukocytes (mil/mm^3^)	6.8 (5.1–9.3)	6.64 (4.9–9)	0.055	9 (6.77–12.1)	9.6 (7.05–12.5)	0.276	8 (5.8–10.95)	7.55 (5.3–10.3)	0.099
Lymphocytes (mil/mm^3^)	0.95 (0.67–1.38)	1.02 (0.70–1.48)	0.001	0.64 (0.44–0.94)	0.68 (0.46–0.96)	0.279	0.81 (0.53–1.26)	0.77 (0.56–1.10)	0.374
Lymphocytes %	14.6 (8.7–22.6)	16.4 (9.9–24.5)	< 0.001	7.1 (4.4–12.0)	6.9 (4.5–11.9)	0.787	10.1 (6–17.4)	10.7 (6.3–18.0)	0.684
Monocytes (mil/mm^3^)	0.50 (0.35–0.68)	0.49 (0.34–0.70)	0.530	0.41 (0.27–0.64)	0.51 (0.34–0.72)	0.010	0.51 (0.34–0.73)	0.48 (0.31–0.69)	0.098
Monocytes %	7.6 (5.3–9.99)	7.6 (5.3–10.1)	0.636	4.8 (3.2–7.015)	5.3 (3.8–7.45)	0.158	6.4 (4.2–9.4)	6.365 (4.1–9.2)	0.396
Neutrophils (mil/mm^3^)	5.00 (3.46–7.39)	4.82 (3.24–6.99)	0.011	7.91 (5.48–10.7)	8.16 (5.77–10.9)	0.548	6.19 (4.25–9.33)	6.07 (3.83–8.67)	0.337
Neutrophils %	76 (66.6–84.4)	74.1 (64.8–82.9)	< 0.001	88.2 (80.2–91.2)	86.6 (80.3–91.1)	0.348	81.7 (72.8–88)	81.6 (72.3–89)	0.798
Basophils (mil/mm^3^)	0.02 (0.01–0.03)	0.02 (0.01–0.03)	0.423	0.02 (0.01–0.03)	0.01 (0.00–0.03)	0.001	0.02 (0.01–0.04)	0.02 (0.01–0.03)	0.162
Basophils %	0.3 (0.2–0.5)	0.3 (0.2–0.5)	0.776	0.2 (0.1–0.3)	0.1 (0.1–0.2)	< 0.001	0.265 (0.1–0.4)	0.27 (0.1–0.4)	0.460
Eosinophils (mil/mm^3^)	0.007 (0–0.036)	0.008 (0–0.039 )	0.471	0 (0–0.016 )	0 (0–0)	0.004	0.002 (0–0.021)	0.001 (0–0.016 )	0.478
Eosinophils %	0.1 (0–0.5)	0.1 (0–0.6)	0.408	0 (0–0.1)	0 (0–0)	0.002	0.04 (0–0.3)	0.01 (0–0.2)	0.503
Red blood cells (mil/mm^3^)	4.52 (4.08–4.87)	4.52 (4.09–4.91)	0.281	4.17 (3.79–4.52)	4.24 (3.80–4.59)	0.280	4.31 (3.84–4.70)	4.34 (3.84–4.72)	0.585
Erythroblasts (mil/mm^3^)	0.001 (0–0.009)	0.002 (0–0.01)	0.088	0.001 (0–0.01)	0 (0–0.01)	0.695	0.001 (0–0.01 )	0.002 (0–0.01)	0.456
Erythroblasts %	0.04 (0–0.1)	0.06 (0–0.1)	0.007	0.01 (0–0.1)	0 (0–0.1)	0.627	0.04 (0–0.1)	0.04 (0–0.1)	0.640
Mean corpuscular hemoglobin concentration (MCHC) (g/dl)	33.4 (32.8–34 )	33.5 (32.9–34.1)	0.022	33.4 (33.0–34.1)	33.5 (33.0–34.1)	0.327	33.1 (32.6–33.7)	33.1 (32.5–33.6)	0.786
Hemoglobin (g/dl)	13.5 (12.3–14.6)	13.6 (12.3–14.7)	0.554	12.6 (11.3–13.7)	12.8 (11.5–1..8)	0.367	13 (11.7–14.2)	1.1 (11.8–14.3)	0.379
Hematocrit (%)	40.4 (36.9–43.5)	40.4 (36.9–43.7)	0.893	37.6 (34.1–40.4)	37.7 (34.6–40.9)	0.392	39.2 (35.4–42.8)	39.4 (35.8–43.1)	0.422
Mean corpuscular volume (MCV) (fl)	90.3 (86.6–93.6)	90 (86.15–93.6)	0.158	90.2 (87.6–93.3)	90.4 (86–93.8)	0.701	91.8 (87.7–95.3)	92.2 (88.5–95.6)	0.413
Platelet count (mil/mm^3^)	188 (143–246)	190 (148–246)	0.610	227 (169–288)	242 (173–312)	0.334	177 (136–230)	172 (131–215)	0.246
Mean platelet volume (MPV) (fl)	9.1 (8.4–9.9)	9.2 (8.5–9.9)	0.197	8.9 (8.275–9.7)	8.9 (8.3–9.6)	0.881	9.2 (8.7–10.2 )	9.4 (8.6–10.1)	0.688
Interleukin-6 (pg/ml)	41.51 (17.18–50)	32.5 (12.6–58.6)	0.161	50 (21.5–82.2)	50 (16.9–94.4)	0.825	50 (36.3–84.2)	50 (25.9–102.2)	0.830
C-reactive protein (mg/l)	8.18 (2.75–15.07)	5.52 (1.64–11.23)	< 0.001	14.56 (5.9–24.61)	8.31 (2.44–15.5)	0.001	11.15 (5.52–1.8)	9.69 (2.48–17.52)	0.065
Procalcitonin (mg/l)	0.13 (0.07–0.32)	0.11 (0.07–0.24)	0.084	0.36 (0.16–0.94)	0.17 (0.09–0.57)	< 0.001	0.24 (0.13–0.73)	0.24 (0.12–0.59)	0.662
Ferritin (ng/ml)	452 (220–1003)	614 (306–1190)	0.007	1277 (583–2727)	1145 (659–2026)	0.971	460 (216–997)	704 (360–1268)	0.054
**Comorbidities/Previous diagnosis**									
Ischemic Cardiopathy	207 (8.1)	180 (6.7)	0.054	27 (8.1)	17 (8.6)	0.880	95 (12.2)	62 (12.4)	0.932
Hypertension	835 (38.2)	877 (35.3)	0.050	115 (38.5)	56 (31.1)	0.108	319 (50.6)	234 (49.1)	0.631
Intermittent claudication	109 (4.3)	97 (3.6)	0.261	19 (5.7)	10 (5.1)	0.854	66 (8.5)	24 (4.8)	0.014
Cerebrovascular disease	292 (11.5)	248 (9.3)	0.013	21 (6.3)	11 (5.6)	0.841	156 (20.0)	106 (11.2)	0.663
Dementia	354 (13.9)	279 (10.4)	< 0.001	21 (6.3)	2 (1.0)	0.002	200 (25.7)	125 (24.9)	0.802
Diabetes	493 (19.4)	588 (22.0)	0.018	72 (21.7)	56 (28.3)	0.096	198 (25.4)	147 (29.3)	0.150
Obesity	366 (4.4)	414 (15.5)	0.251	69 (20.8)	55 (27.8)	0.090	96 (12.3)	82 (16.4)	0.048
Chronic obstructive pulmonary disease (COPD)	367 (14.4)	375 (14.0)	0.717	52 (15.7)	31 (15.7)	1.000	137 (17.6)	96 (19.2)	0.506
Previous pneumonia	381 (15.0)	230 (8.6)	< 0.001	76 (22.9)	20 (10.1)	< 0.001	117 (15.0)	47 (9.4)	0.004
Malignancy	132 (5.2)	124 (4.6)	0.362	16 (4.8)	8 (4.0)	0.824	57 (7.3)	32 (6.4)	0.587
**Previous Treatment**									
Gastric secretion inhibitors	1045 (41)	970 (36.3)	< 0.001	124 (37.3)	72 (36.4)	0.856	454 (58.3)	273 (54.5)	0.187
Antidiabetics	408 (16)	471 (17.6)	0.128	61 (18.4)	56 (28.3)	0.008	164 (21.1)	114 (22.7)	0.479
Antithrombotics	739 (29)	688 (25.7)	0.007	78 (23.5)	43 (21.7)	0.669	359 (46.1)	219 (43.7)	0.431
Beta-blockers	400 (15.7)	364 (13.6)	0.034	55 (16.6)	27 (15.6)	0.367	177 (22.7)	105 (21)	0.466
Potassium-sparing diuretics	115 (4.5)	94 (3.5)	0.07	9 (2.7)	5 (2.5)	1.000	61 (7.8)	20 (4)	0.007
Anxiolytics	276 (10.8)	237 (8.9)	0.019	46 (13.9)	23 (11.6)	0.511	184 (23.6)	102 (20.4)	0.200
Antidementia drugs	41 (1.6)	37 (1.4)	0.559	11 (3.3)	1 (0.5)	0.057	88 (11.3)	68 (13.6)	0.262
Nutritional supplements	194 (7.6)	188 (7)	0.198	9 (2.7)	1 (0.5)	0.088	78 (10)	31 (6.2)	0.021

ICU: intensive care unit; IQR: interquartile range; NA: not applicable.

Patients were grouped as hospitalized patients (total number of patients), admitted to the ICU, and deceased. Laboratory and clinical variables were baseline values and are presented as the median and interquartile range (IQR). Units are shown in parentheses. Values of sex, comorbidities and previous treatment are displayed as n and the percentage of total patients (%).

Compared to the second wave, patients hospitalized in the first wave showed signs of greater disease severity, including a higher heart rate, higher temperature, lower oxygen saturation, and higher levels of creatinine, C-reactive protein, LDH, and fibrinogen (Table 1). Other parameters of disease severity, such as neutrophilia and lymphopenia, indicated worse disease in patients infected in the first wave than those infected in the second wave.

Among patients admitted to the ICU, those in the first wave were significantly older (70 years [60–76] vs. 62 years [54–72]) and had a higher rate of dementia compared to those admitted to the ICU in the second wave. Patients in the first wave also took antidiabetic drugs less frequently and exhibited higher levels of both clinical and analytical markers of serious illness compared to patients in the second wave (Table 1).

Unlike patients who were hospitalized or admitted to the ICU, patients who died in the first wave were younger than those who died in the second wave (85 years [77–90] vs. 87 years [81–91]; Table 1). Moreover, they were less obese in the first wave than in the second wave. Nevertheless, patients who died in the first wave had greater disease severity parameters than those who died in the second wave.

### Mortality risk factors

The univariate analysis (Table 2) showed that mortality was best predicted in the first wave by urea (AUC = 0.81), age (AUC = 0.79), D-dimer (AUC = 0.73), and creatinine (AUC = 0.72). The best predictors of mortality in the second wave were age (AUC = 0.82), urea (AUC = 0.77), D-dimer (AUC = 0.71), and lymphocytes (AUC = 0.70).

**Table 2:** Univariate analysis of potential predictors of death in the two pandemic waves, Aragon, Spain, February–September, 2020.

Variable	First wave			p value adjusted	Second wave			p value adjusted
	AUC	OR (95% CI)	p value		AUC	OR (95% CI)	p value	
**Univariant Model**								
Sex (Male: Female)	0.512	0.910 (0.789–1.05)	0.279	0.976	0.5	0.997 (0.846–1.174)	0.976	1.0
Age (years)	0.79	1.087 (1.08–1.095)	< 0.001	< 0.001	0.821	1.093 (1.085–1.102)	< 0.001	< 0.001
**Emergency room**								
Systolic pressure (mmg Hg)	0.557	0.992 (0.988–0.995)	< 0.001	0.006	0.499	0.999 (0.995–1.003)	n.s	n.s
Diastolic pressure (mmHg)	0.595	0.977 (0.971–0.983)	< 0.001	< 0.001	0.584	0.974 (0.967–0.981)	< 0.001	< 0.001
Temperature (ºC)	0.504	1.015 (0.975–1.057)	n.s	n.s	0.543	1.032 (0.979–1.099)	n.s	n.s
Oxygen saturation (%)	0.609	0.905 0.888–0.921)	< 0.001	< 0.001	0.589	0.89 (0.868–0.913)	< 0.001	< 0.001
Capillary blood glucose (mg/dl)	0.614	1.006 (1.003–1.009)	< 0.001	0.017	0.656	1.003 (1.001–1.005)	n.s	n.s
**Laboratory**								
Glucose (mg/dl)	0.603	1.006 (1.004–1.008)	< 0.001	< 0.001	0.6265	1.007 (1.005–1.009)	< 0.001	< 0.001
Creatinine (mg/dl)	0.716	2.353 (2.019–2.762)	< 0.001	< 0.001	0.696	2.36 (2.009–2.793)	< 0.001	< 0.001
Urea (g/l)	0.81	16.932 (12.12–24.01)	< 0.001	< 0.001	0.7676	8.396 (6.181–11.538)	< 0.001	< 0.001
Chloride (mmol/l)	0.602	1.069 (1.051–1.087)	< 0.001	< 0.001	0.5413	1.043 (1.0258–1.0607)	< 0.001	0.002
Potassium (mmol/l)	0.574	1.645 (1.365–1.986)	< 0.001	0.001	0.5275	1.235 (0.99–1.54)	n.s	n.s
Alanine aminotransferase (ALT) (U/l)	0.596	0.993 (0.989–0.996)	0.002	n.s	0.6151	0.993 (0.989–0.997)	0.005	n.s
Lactate dehydrogenase (LDH) (U/l)	0.589	1.002 (1.001–1.003)	< 0.001	< 0.001	0.5952	1.003 (1.002–1.004)	< 0.001	< 0.001
Prothrombin activity (APT) (%)	0.617	0.979 (0.974–0.984)	< 0.001	< 0.001	0.6071	0.982 (0.978–0.987)	< 0.001	< 0.001
International normalized ratio-prothrombin time (INR-PT)	0.616	1.527 (1.314–1.82)	< 0.001	0.008	0.6047	1.398 (1.202–1.641)	< 0.001	0.016
Active partial thromboplastin time (RATIO-APTT) (seconds)	0.534	2.954 (1.839–4.792)	< 0.001	0.001	0.5115	1.403 (0.8801–2.190)	n.s	n.s
D-Dimer (microgr/l)	0.728	1 (1–1)	< 0.001	0.018	0.7127	1 (1–1.0001)	< 0.001	0.002
Leukocytes (mil/mm^3^)	0.632	1.136 (1.108–1.167)	< 0.001	< 0.001	0.5908	1.085 (1.061–1.111)	< 0.001	< 0.001
Lymphocytes (mil/mm^3^)	0.605	0.721 (0.608–0.846)	0.001	0.04	0.6626	0.392 (0.315–0.483)	< 0.001	< 0.001
Lymphocytes (%)	0.674	0.944 (0.933–0.955)	< 0.001	< 0.001	0.7002	0.926 (0.913–0.938)	< 0.001	< 0.001
Monocytes (mil/mm^3^)	0.518	1.653 (1.299–2.125)	< 0.001	0.029	0.4765	1.06 (0.835–1.302)	n.s	n.s
Monocytes (%)	0.596	0.95 (0.925–0.974)	< 0.001	0.032	0.6152	0.915 (0.887–0.943)	< 0.001	< 0.001
Neutrophils (mil/mm^3^)	0.647	1.157 (1.126–1.19)	< 0.001	< 0.001	0.6313	1.127 (1.099–1.157)	< 0.001	< 0.001
Neutrophils (%)	0.663	1.042 (1.034–1.052)	< 0.001	< 0.001	0.6899	1.059 (1.049–1.07)	< 0.001	< 0.001
Basophils (mil/mm^3^)	0.504	100.431 (2.111–4611.052)	0.048	n.s	0.5271	0.177 (0.003–6.835)	n.s	n.s
Basophils (%)	0.571	0.648 (0.445–0.923)	n.s	n.s	0.5874	0.380 (0.247–0.572)	< 0.001	0.008
Eosinophils (mil/mm^3^)	0.587	0.29 (0.058–1.288)	n.s	n.s	0.6072	0.062 (0.011–0.292)	0.0055	n.s
Eosinophils (%)	0.599	0.875 (0.78–0.972)	0.046	n.s	0.6178	0.717 (0.618–0.819)	< 0.001	0.005
Red blood cells (mill/mm^3^)	0.625	0.502 (0.427–0.588)	< 0.001	< 0.001	0.6034	0.569 (0.483–0.669)	< 0.001	< 0.001
Erythroblasts (mil/mm^3^)	0.538	666,455,985.639 (559.172–5875,529,981,928,593)	0.029	n.s	0.5306	1.422 (0.132–8.167)	n.s	n.s
Mean corpuscular hemoglobin concentration (MCHC) (g/dl)	0.641	0.564 (0.502–0.633)	< 0.001	< 0.001	0.6502	0.593 (0.53–0.662)	< 0.001	< 0.001
Hemoglobin (g/dl)	0.616	0.8 (0.757–0.846)	< 0.001	< 0.001	0.5796	0.866 (0.82–0.914)	< 0.001	0.001
Hematocrit (%)	0.588	0.946 (0.929–0.964)	< 0.001	< 0.001	0.5524	0.969 (0.951–0.988)	0.006	n.s
Mean corpuscular volume (MCV) (fl)	0.604	1.064 (1.046–1.083)	< 0.001	< 0.001	0.6277	1.073 (1.054–1.093)	< 0.001	< 0.001
Platelet count (mil/mm^3^)	0.565	0.997 (0.996–0.998)	< 0.001	0.003	0.596	0.996 (0.995–0.998)	< 0.001	< 0.001
Mean platelet volume (MPV) (fl)	0.566	1.213 (1.117–1.317)	< 0.001	0.005	0.5504	1.165 (1.066–1.273)	0.005	n.s
Interleukin-6 (pg/ml)	0.676	1.008 (1.005–1.012)	< 0.001	0.001	0.6659	1.004 (1.002–1.006)	< 0.001	0.013
C-reactive protein (mg/l)	0.625	1.045 (1.03–1.06)	< 0.001	< 0.001	0.6351	1.071 (1.051–1.091)	< 0.001	< 0.001
Procalcitonin (mg/l)	0.73	1.075 (1.029–1.135)	0.018	n.s	0.7363	1.318 (1.155–1.546)	0.002	n.s
**Comorbidities**								
Malignancy	0.515	1.774 (1.311–2.391)	0.002	n.s	0.511	1.562 (1.091–2.2)	0.036	n.s
Vitamin B12 and folate deficiency	0.522	2.424 (1.773–3.318)	< 0.001	< 0.001	0.52	2.393 (1.666–3.4)	< 0.001	0.003
Iron-deficiency anemia	0.564	2.319 (1.94–2.772)	< 0.001	< 0.001	0.55	1.902 (1.558–2.316)	< 0.001	< 0.001
Hemostatic alterations	0.547	2.45 (1.977–3.037)	< 0.001	< 0.001	0.538	2.315 (1.789–2.981)	< 0.001	< 0.001
Eosinophilia	0.506	1.95 (1.18–3.203)	0.027	n.s	0.506	2.226 (1.171–4.069)	0.033	n.s
Asymptomatic hyperuricemia	0.518	1.22 (1.034–1.438)	0.048	n.s	0.509	1.103 (0.911–1.331)	n.s	n.s
Myelodysplastic syndromes	0.505	2.664 (1.313–5.489)	0.023	n.s	0.5	1.05 (0.314–2.791)	n.s	n.s
Acute pancreatitis	0.512	1.771 (1.258–2.483)	0.006	n.s	0.502	1.15 (0.723–1.767)	n.s	n.s
Ischemic cardiomyopathy	0.53	2.106 (1.648–2.688)	< 0.001	< 0.001	0.535	2.441 (1.852–3.2)	< 0.001	< 0.001
Heart failure	0.547	3.457 (2.679–4.474)	< 0.001	< 0.001	0.551	3.454 (2.645–4.498)	< 0.001	< 0.001
Atrial fibrillation	0.549	2.899 (2.302–3.656)	< 0.001	< 0.001	0.54	2.486 (1.919–3.205)	< 0.001	< 0.001
Pulmonary hypertension	0.505	3.7 (1.605–9.062)	0.012	n.s	0.501	2.806 (0.536–12.537)	n.s	n.s
Non rheumatic heart disease aortic stenosis	0.509	1.866 (1.23–2.817)	0.013	n.s	0.5	1.05 (0.562–1.836)	n.s	n.s
Atrial fibrillation	0.526	1.827 (1.439–2.316)	< 0.001	0.001	0.534	2.355 (1.788–3.082)	< 0.001	< 0.001
Hypertension	0.596	2.225 (1.918–2.586)	< 0.001	< 0.001	0.619	2.74 (2.297–3.277)	< 0.001	< 0.001
Stroke	0.562	3.075 (2.492–3.798)	< 0.001	< 0.001	0.573	3.881 (3.076–4.89)	< 0.001	< 0.001
Intermittent claudication	0.53	3.775 (2.709–5.303)	< 0.001	< 0.001	0.508	1.483 (0.983–2.187)	n.s	n.s
Cognitive impairment or dementia	0.585	3.653 (3.006–4.445)	< 0.001	< 0.001	0.589	4.36 (3.497–5.431)	< 0.001	< 0.001
Non-streptococcal tonsillitis	0.507	0.352 (0.158–0.69)	0.018	n.s	0.511	0.256 (0.097–0.549)	0.009	n.s
Acute bronchitis	0.539	1.638 (1.374–1.95)	< 0.001	< 0.001	0.545	1.742 (1.43–2.116)	< 0.001	< 0.001
Flu	0.524	0.213 (0.121–0.348)	< 0.001	< 0.001	0.517	0.338 (0.181–0.578)	0.002	n.s
Respiratory infections	0.512	1.72 (1.241–2.373)	0.006	n.s	0.511	1.617 (1.107–2.317)	0.032	n.s
Chronic obstructive pulmonary disease (COPD)	0.522	1.404 (1.155–1.704)	0.004	n.s	0.531	1.596 (1.284–1.974)	< 0.001	0.016
Obstructive sleep apnea	0.515	1.475 (1.142–1.897)	0.012	n.s	0.509	1.328 (0.967–1.798)	n.s	n.s
Obesity	0.516	0.768 (0.621–0.945)	0.039	n.s	0.505	1.082 (0.864–1.348)	n.s	n.s
Diabetes mellitus	0.545	1.744 (1.467–2.072)	< 0.001	< 0.001	0.546	1.64 (1.362–1.969)	< 0.001	0.001
Vitamin D deficiency	0.539	1.63 (1.369–1.939)	< 0.001	< 0.001	0.545	1.698 (1.401–2.054)	< 0.001	< 0.001
Gout	0.51	1.46 (1.076–1.97)	0.039	n.s	0.516	1.746 (1.255–2.398)	0.005	n.s
Hyperlipidemia	0.512	1.107 (0.959–1.277)	n.s	n.s	0.535	1.334 (1.131–1.572)	0.004	n.s
**Previous treatments**								
Antiulcer drugs	0.625	2.794 (2.415–3.235)	< 0.001	< 0.001	0.614	2.578 (2.183–3.046)	< 0.001	< 0.001
Laxatives	0.51	2.054 (1.361–3.091)	0.004	n.s	0.514	3.456 (2.081–5.689)	< 0.001	0.002
Antidiabetic drugs	0.537	1.694 (1.406–2.038)	< 0.001	< 0.001	0.533	1.522 (1.243–1.857)	< 0.001	0.024
Vitamins: A, D, E	0.517	1.322 (1.078–1.616)	0.023	n.s	0.534	1.741 (1.389–2.17)	< 0.001	0.002
Calcium	0.517	1.576 (1.224–2.021)	0.003	n.s	0.518	1.779 (1.301–2.404)	0.002	n.s
Antithrombotic therapy	0.624	3.168 (2.719–3.693)	< 0.001	< 0.001	0.611	2.845 (2.395–3.378)	< 0.001	< 0.001
Oral iron	0.537	2.659 (2.06–3.436)	< 0.001	< 0.001	0.538	3.021 (2.256–4.027)	< 0.001	< 0.001
Vitamin B12 and folic acid	0.557	2.544 (2.082–3.109)	< 0.001	< 0.001	0.546	2.076 (1.666–2.578)	< 0.001	< 0.001
Digoxin and other cardiac glycosides	0.523	1.243 (1.066–1.448)	0.019	n.s	0.534	1.37 (1.152–1.627)	0.003	n.s
Vasodilators with venodilator action: nitroglycerin	0.523	3.952 (2.669–5.928)	< 0.001	< 0.001	0.521	2.816 (1.932–4.067)	< 0.001	< 0.001
Prostaglandins	0.503	1.873 (0.939–3.688)	n.s	n.s	0.506	1.974 (1.105–3.399)	0.045	n.s
Thiazide and thiazide-like diuretics (chlorthalidone)	0.507	2.054 (1.266–3.324)	0.014	n.s	0.507	2.056 (1.173–3.488)	0.029	n.s
Loop diuretic (furosemide, torasemide)	0.594	3.524 (2.936–4.234)	< 0.001	< 0.001	0.589	3.122 (2.565–3.794)	< 0.001	< 0.001
Potassium sparing diuretics	0.524	2.661 (1.938–3.661)	< 0.001	< 0.001	0.504	1.244 (0.799–1.88)	n.s	n.s
Chronic venous disease treatment	0.506	2.832 (1.458–5.597)	0.01	n.s	0.507	3.039 (1.501–5.997)	0.008	n.s
Varicose veins treatment	0.502	1.892 (0.735–4.761)	n.s	n.s	0.507	3.125 (1.591–6.004)	0.004	n.s
Beta-blockers	0.551	2.038 (1.693–2.451)	< 0.001	< 0.001	0.545	1.965 (1.587–2.425)	< 0.001	< 0.001
Dihydropyridine calcium channel blockers	0.521	1.611 (1.272–2.034)	< 0.001	0.031	0.534	2.044 (1.59–2.612)	< 0.001	< 0.001
Nondihydropyridine calcium channel blockers (verapamil, diltiazem)	0.511	2.611 (1.658–4.131)	< 0.001	0.02	0.509	2.359 (1.383–3.926)	0.007	n.s
Angiotensin-converting enzyme inhibitors	0.533	1.833 (1.482–2.264)	< 0.001	< 0.001	0.527	1.698 (1.327–2.157)	< 0.001	0.015
Angiotensin II receptor blockers	0.537	1.492 (1.267–1.754)	< 0.001	0.002	0.538	1.514 (1.256–1.82)	< 0.001	0.011
Medical treatment of benign prostatic hyperplasia	0.55	2.561 (2.07–3.169)	< 0.001	< 0.001	0.535	2.054 (1.603–2.619)	< 0.001	< 0.001
Systemic glucocorticoids	0.51	1.807 (1.244–2.614)	0.009	n.s	0.512	2.31 (1.468–3.569)	0.002	n.s
Oral contraceptive	0.506	2.832 (1.458–5.597)	0.01	n.s	0.507	3.549 (1.721–7.21)	0.003	n.s
Pharmacologic urate-lowering therapy	0.537	2.39 (1.885–3.03)	< 0.001	< 0.001	0.527	1.977 (1.501–2.585)	< 0.001	0.002
Bisphosphonates	0.523	2.807 (2.009–3.935)	< 0.001	< 0.001	0.507	1.487 (0.967–2.229)	n.s	n.s
Analgesics	0.559	2.231 (1.861–2.675)	< 0.001	< 0.001	0.551	2.043 (1.66–2.507)	< 0.001	< 0.001
Antiepileptics	0.532	1.843 (1.487–2.283)	< 0.001	< 0.001	0.544	2.348 (1.845–2.975)	< 0.001	< 0.001
Antiparkinson drugs	0.52	3.221 (2.193–4.768)	< 0.001	< 0.001	0.518	3.331 (2.141–5.142)	< 0.001	< 0.001
Antipsychotics	0.563	3.28 (2.642–4.078)	< 0.001	< 0.001	0.564	3.419 (2.697–4.324)	< 0.001	< 0.001
Anxiolytics	0.552	2.035 (1.696–2.44)	< 0.001	< 0.001	0.53	1.519 (1.229–1.868)	0.001	0.043
Hypnotic therapy	0.53	2.028 (1.599–2.568)	< 0.001	< 0.001	0.543	2.699 (2.081–3.486)	< 0.001	< 0.001
Antidepressants	0.582	2.383 (2.028–2.801)	< 0.001	< 0.001	0.59	2.578 (2.153–3.085)	< 0.001	< 0.001
Psychostimulants	0.509	2.764 (1.611–4.789)	0.002	n.s	0.507	2.308 (1.277–4.042)	0.016	n.s
Antidementia drugs	0.535	2.821 (2.155–3.699)	< 0.001	< 0.001	0.549	4.024 (3.02–5.352)	< 0.001	< 0.001
Chronic obstructive pulmonary disease treatments	0.525	1.714 (1.362–2.152)	< 0.001	0.005	0.536	2.032 (1.592–2.578)	< 0.001	< 0.001
Antiglaucoma medications	0.526	1.991 (1.549–2.556)	< 0.001	< 0.001	0.525	1.968 (1.481–2.595)	< 0.001	0.004
Nutritional supplements	0.538	4.538 (3.29–6.321)	< 0.001	< 0.001	0.523	4.137 (2.713–6.3)	< 0.001	< 0.001

OR: Odds ratio; CI: confidence interval; n.s.: Not Significant. P value adjusted: adjusted p-values for multiple comparisons using the Holm method.

The multivariate analysis (Table 3) showed that, in both pandemic waves, age (OR = 1.072), elevated temperature (OR = 1.300), Urea (OR = 2.982), Potasisum (1.705), LDH (OR = 1.002), monocytes (OR = 2.231) and neutrophils (OR = 1.037) levels, malignancy (OR = 2.952), and taking vasodilators (OR = 3.490), potassium sparing diuretics (OR = 2.315), antipsychotics (OR = 3.247), antidepressants (OR = 1.593), antidementia drugs (OR = 2.125) and nutritional supplements (OR = 2.433) before hospitalization were independent risk factors associated with mortality. Conversely, normal oxygen saturation (OR = 0.949) and platelet levels (OR = 0.990) were protective factors.

**Table 3:** Multivariate analysis of potential predictors of death in the two pandemic waves, Aragon, Spain, February–September, 2020.

Variable	Predictor		Interaction term (ref: 1st wave)	
	OR (95% CI)	p-value	OR (95%CI)	p-value
Age (years)	1.072 (1.054–1.090)	< 0.001	0.999 (0.983–1.023)	0.939
Temperature (ºC)	1.300 (1.078–1.566)	0.006	0.943 (0.779–1.141)	0.546
Oxygen saturation (%)	0.949 (0.911–0.989)	0.013	1.044 (0.978–1.115)	0.191
Urea (g/l)	2.982 (1.741–5.109)	< 0.001	0.656 (0.308–1.439)	0.300
Potassium (mmol/l)	1.705 (1.250–2.326)	< 0.001	0.687 (0.431–1.095)	0.115
Alanine aminotransferase (ALT) (U/l)	0.990 (0.984–0.997)	0.003	1.004 (0.978–1.115)	0.366
Lactate dehydrogenase (LDH) (U/l)	1.002 (1.001–1.003)	0.004	1.001 (0.999–1.003)	0.224
Monocytes (mil/mm^3^)	2.231 (1.375–3.620)	0.001	0.488 (0.266–0.896)	**0.021**
Neutrophils (%)	1.037 (1.019–1.055)	< 0.001	0.994 (0.969–1.019)	0.642
Platelet count (mil/mm^3^)	0.995 (0.992–0.997)	< 0.001	1.001 (0.998–1.005)	0.322
Malignancy	2.952 (1.267–6.877)	0.012	0.667 (0.214–2.077)	0.485
Vasodilators	3.490 (1.290–9.405)	0.013	0.543 (0.141–2.085)	0.374
Potassium sparing diuretics	2.315 (1.109–4.832)	0.025	0.171 (0.048–0.605)	**0.006**
Antipsychotics	3.247 (1.281–8.231)	0.013	0.331 (0.087–1.261)	0.105
Antidepressants	1.593 (1.067–.2.378)	0.022	0.676 (0.370–1.235)	0.203
Antidementia drugs	2.125 (1.092–4.137)	0.026	0.480 (0.182–1.262)	0.136
Nutritional supplements	2.433 (1.063–5.569)	0.035	0.884 (0.231–3.388)	0.857

Most of these factors shown no statistically significant between waves, we only found that monocytes (OR = 0.488) and taking potassium sparing (OR = 0.171) diuretics decreased their risk of mortality in the second wave.

### Longitudinal analysis

**Figure 5:**
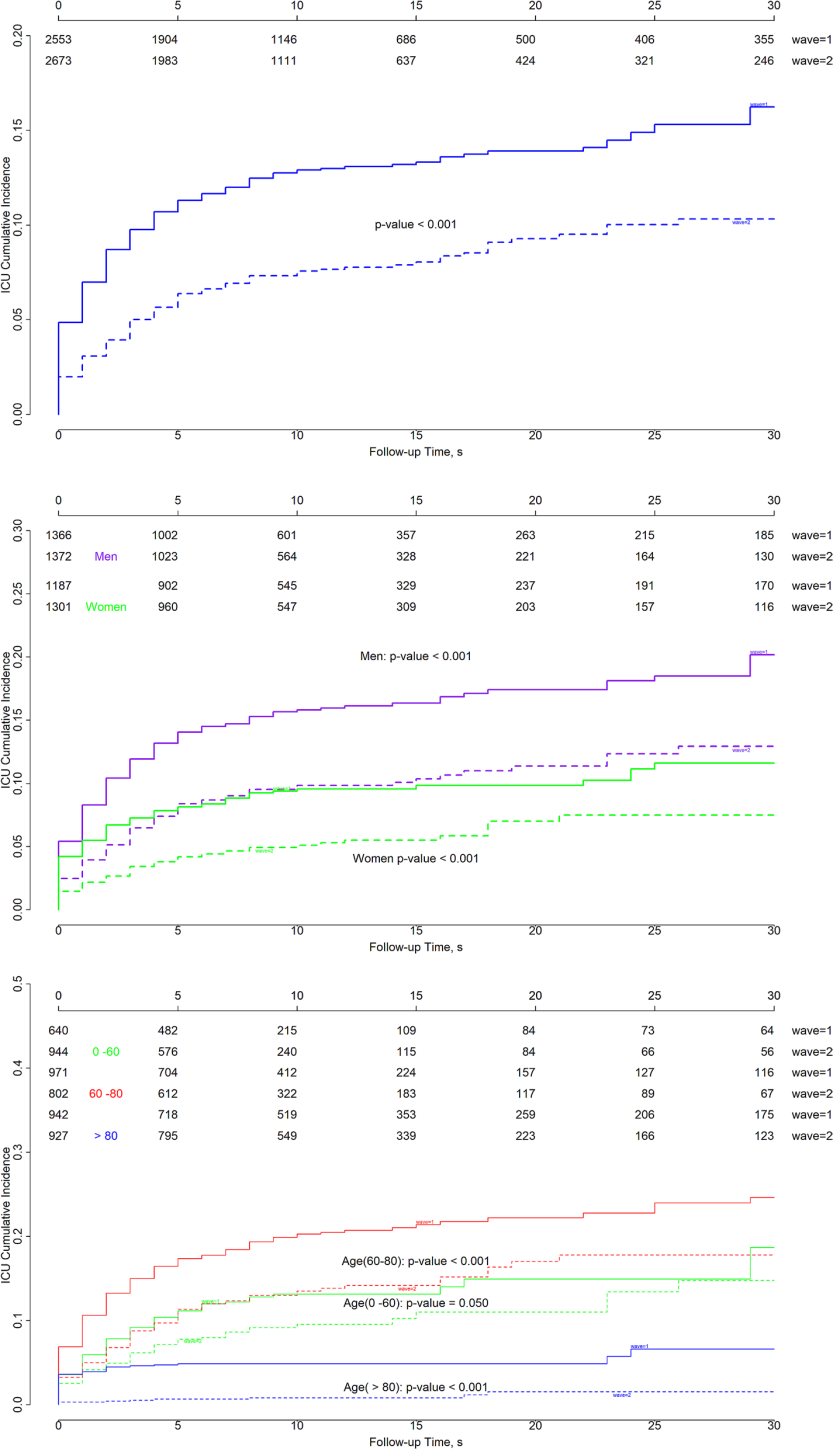
Cumulative incidence of ICU admissions for all patients (*top),* patients stratified by sex *(middle*), and patients stratified by age (*bottom)* in Aragon, Spain, February-September 2020. By sex: *purple* indicates men, *green* indicates women. By age: *green* indicates 0–60 years, *red* 60–80 years, and *blue* > 80 years. *Solid lines*: first wave; *dashed lines*: second wave. The p-values corresponds to the Gray test to compare survival curves between waves for groups stratified by sex or age.

The cumulative incidence of ICU admission (Fig. 5) was significantly different between the two waves (p < 0.001). The probability of ICU admission ranged from 13% at 10 days to 16% at 30 days during the first wave, and from 8% at 10 days to 10% at 30 days during the second wave. Stratifying by sex, the ICU admission probability among men was 16% to 20% during the first wave and 10% to 13% during the second wave (p < 0. 001). The probability of ICU admission was lower among women; it ranged from 10 to 12% during the first wave and from 5 to 7% during the second wave (p < 0.001).

When stratified by age, we found that the need for ICU admission was significantly different between waves for the 0–60 years group (p = 0.05), the 60–80 years group (p < 0.001), and the > 80 years group (p < 0.001). For these three age groups, the rates of ICU admission were 13%-19%, 18%-25%, and 5%-7%, respectively, in the first wave, and 10%-15%, 14%-18%, and 1%-2%, respectively, in the second wave.

**Figure 6:**
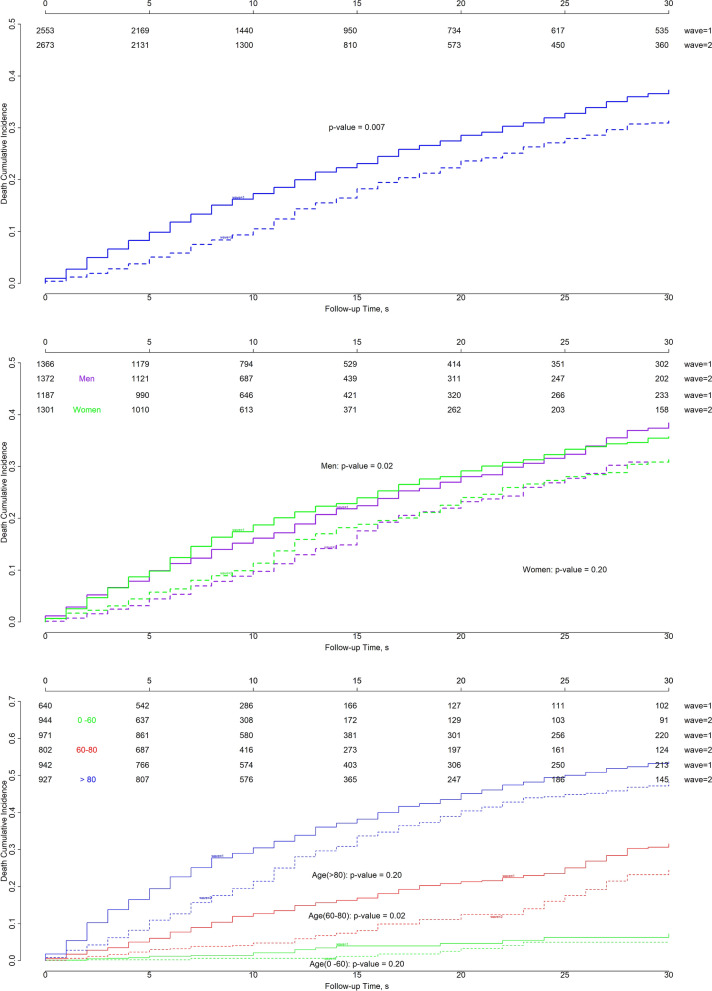
Cumulative incidence of death for all patients (*top)*, patients stratified by sex (*middle*), and patients stratified by age (*bottom)* in Aragon, Spain, February-September 2020. By sex: *purple* indicates men, *green* indicates women. By age: *green* indicates 0–60 years, *red* 60–80 years, and *blue* > 80 years. *Solid lines*: first wave; *dashed lines*: second wave. The p-values corresponds to the Gray test to compare survival curves between waves for groups stratified by sex or age.

Overall survival was significantly different between waves (p = 0.007; Fig. 6). The probability of death ranged from 18% at 10 days to 37% at 30 days in the first wave, and from 11% at 10 days to 32% at 30 days in the second wave. In the first wave, mortality was greater for patients with either short or long hospitalizations. For men, mortality ranged from 16 to 38% in the first wave and from 10 to 32% in the second wave (p = 0.02). For women, mortality ranged from 19 to 36% in the first wave and from 11 to 32% in the second wave (p = 0.2). In both waves, mortality was greater among men than among women.

Mortality increased with age in both waves. However, overall survival was significantly different between waves in the 60–80 year group (p = 0. 02), but not in the 0–60 year or > 80 year groups (p = 0. 20). In the first wave, the probability of death at 10–30 days was 2%-7%, 13%-32%, and 30%-54% for hospitalized patients aged 0–60 years, 60–80 years, and > 80 years, respectively. Notably, the probability of dying was lower in the youngest group than in the two older groups. Remarkably, the probability of death was higher for longer hospitalization periods, particularly among the oldest patients. In the second wave, the probability of death was 1%-5%, 5%-24%, and 21%-48% for the three age groups, respectively. These values were lower than those observed in the first wave, though the trends were similar.

### Viral genome analysis

We analyzed 236 virus samples from the first wave and 56 from the second wave. The distribution of the D614G spike protein mutation was different between waves. It was present in 66% of viruses studied in the first wave and 100% of viruses studied in the second wave. According to the GISAID classification, 32% of the viruses analyzed during the first wave belonged to clades S (characterized by the L84S mutation in the NS8 protein) and V (with the G251V mutation in the NS3 protein), but these virus strains disappeared in the second wave. In contrast, 98.2% of viruses studied in the second wave belonged to clade G (characterized by the D614G spike protein mutation) and 1.8% belonged to clade GR (with the D614G spike protein mutation and the G204R mutation in the nucleocapsid protein).

## Discussion

Aragon was one of the first regions in Europe to experience the emergence of the second pandemic wave of SARS-CoV-2 infection. This experience allowed early analyses and comparisons of the two waves, which could shed light on the future evolution of the pandemic.

Although the number of hospitalized patients was similar in both waves, we found significant differences in the increase in cases over time. The intensity of the first surge was very difficult for the healthcare system to manage, which led to its collapse or near-collapse. However, that degree of intensity did not occur during the second wave.

Another difference between the two waves was that the hospitalized patients were younger in the second wave than in the first wave. This difference was probably due to the greater exposure of younger people in the community in the second wave and the greater protection of older people, who were more concerned about the perils of the disease.

Compared to the first wave, the second wave had less marked clinical (high fever) and analytical (lymphopenia, elevated D-dimer) predictors of worse outcomes^19,20^. Moreover, age-related comorbidities, such as cerebrovascular disease and dementia, were less prevalent in the second wave than in the first wave. Interestingly, diabetes was more prevalent in the second wave.

Patients transferred to the ICU had somewhat different characteristics between waves. Compared to the first wave, patients in the second wave were younger and more frequently took antidiabetic drugs. In addition, the frequency of cognitive impairment was lower in the second wave. It is likely that patients with these features were also infected in the first wave but the number was obscured by the larger number of older and seriously ill patients. Older and seriously ill patients were less numerous in the second wave than in the first wave. However, although patients who required hospitalization and ICU care were younger in the second wave than in the first wave, the median age of death was higher in the second wave.

The most relevant finding in our study was that the overall 30-day mortality of hospitalized patients declined during the second wave. This significant decline in mortality affected essentially all patients aged 60 to 80 years old. A potential cause for this finding was that hospitalized patients had less severe parameters overall in the second wave than in the first wave. These parameters included vital signs and the clinical inflammation markers that serve as prognostic factors for severity^21–23^. Another explanation for the difference in mortality between waves could be improvements in the clinical management of patients. Although no antiviral drugs have clearly increased survival rates in patients with COVID-19, other advances in the management of patients with more severe infections have been associated with improved outcomes^24–26^. In addition, unlike the rapid increase observed in the first wave, during the second wave, the number of cases increased gradually. Therefore, although the health system had been overloaded, health resource use increased gradually, which prevented a system collapse. As such, this situation may be associated with better outcomes.

Finally, we could not rule out the possibility that mutations in the virus may have reduced the virulence of SARS-CoV-2. We analyzed viral genomes from the first and second waves and found that the spike 614G mutation, which was abundant only at the end of the first wave, was present in all genomes isolated in the second wave. These findings have been described throughout the country^27^. Initially, this mutation was associated with greater disease severity^28^. However, a recent study indicated that, although the G614 variant is related to greater infectivity and higher viral loads, there is no evidence that it is associated with disease severity^29^.

This study has some limitations. In the first 2 months of the pandemic, there was a significant shortage of diagnostic tests. This situation prevented an overall comparison between pandemic waves. Due to these limitations, we focused the comparison on patients admitted to the hospital. In this environment, there has never been a shortage of diagnostic tests. Other limitations of this study were primarily due to its retrospective nature and the data source (i.e., electronic medical records). Furthermore, this study included data from the entire region, and different hospitals may have employed different management criteria and different resource allocations. However, some of these limitations are compensated for by the high number of patients included.

In conclusion, patients in the first wave have worse clinical characteristics, and consequently the cumulative ICU admission and mortality were higher than those of the second wave. Regarding independent risk factor of mortality, they were similar in both waves, showing only differences in monocytes and potassium diuretics savers variables. Our study show differences between waves in our cohort and we can expect that vaccination also influence in the future for the trajectory of hospitalized patients by COVID-19 infection.

## Data Availability

The clinical and demographic data analyzed were retrieved through the *BIGAN Gestion Clinica* platform of the Aragón Department of Health, which contains the information from the Aragón Healthcare Records Database. Whole genome sequences of SARS-CoV-2 were retrieved from the database established by the global initiative on sharing all influenza data (GISAID).
